# Occupational exposure, attitude to HIV-positive patients and uptake of HIV counselling and testing among health care workers in a tertiary hospital in Nigeria

**DOI:** 10.1080/17290376.2017.1398104

**Published:** 2017-11-13

**Authors:** Modupe O. Onadeko, Mary O. Balogun, Olanrewaju O. Onigbogi, Folashade O. Omokhodion

**Affiliations:** ^a^ FWACP, MPH, MD, Professor of Community Medicine, Department of Community Medicine, University College Hospital Ibadan, Ibadan, Oyo State, Nigeria,; ^b^ MBBS, MPH, FWACP, DOccMed, Lecturer and Consultant Community Physician, Department of Community Medicine, University College Hospital Ibadan, Ibadan, Oyo State, Nigeria; ^c^ MBBS, MPH, FMCPH, Lecturer and Consultant Community Physician, Department of Community Health and Primary Care, College of Medicine, University of Lagos, Lagos State, Nigeria; ^d^ MBBS, MSc, PhD, FWACP, FFOM, FFPH, Professor and Consultant Community Physician, Department of Community Medicine, University College Hospital Ibadan, Ibadan, Oyo State, Nigeria.

**Keywords:** *health care workers*, *occupational exposure*, *attitude to HIV patients*, *HIV counselling and testing*

## Abstract

Health care workers (HCWs) are at risk of occupational exposure to HIV. Their attitude to HIV-positive patients influences patients’ willingness and ability to access quality care. HIV counselling and testing (HCT) services are available to inform HCWs and patients about their status. There is little information about HCT uptake and attitude to HIV-positive patients among HCWs in tertiary health facilities in Nigeria. The aim of this study was to determine occupational exposure and attitude to HIV-positive patients and level of uptake of HCT services among HCWs in a tertiary hospital in Nigeria. A cross-sectional design was utilized. A total of 977 HCWs were surveyed using semi-structured, self-administered questionnaires. Nurses and doctors comprised 78.2% of the respondents. Their mean age was 35 ± 8.4 years. Almost half, 47.0%, reported accidental exposure to blood and body fluids (BBFs) in the preceding year. The main predictor of accidental exposure to BBFs in the last year was working in a surgical department, OR = 1.7, 95% CI (1.1–2.6). HCWs aged <40 years, OR = 5.5, 95% CI (1.9–15.9), who had worked for >5 years, OR = 3.6, 95% CI (1.4–9.3) and who work in nursing department, OR = 6.8, 95% CI (1.7–27.1) were more likely to be exposed to BBFs. Almost half, 52.9%, had accessed HCT services. Predictors for HCT uptake were age <40 years OR = 1.6, 95% CI (1.1–2.4), having worked for >5 years OR = 1.5, 95% CI (1.03–2.2) and working in medical department OR = 1.7, 95% CI (1.1–2.8). Respondents in nursing departments were more likely to require routine HIV test for all patients, OR = 3.9, 95% CI (2.4–6.2). HCWs in the laboratory departments were more likely to believe that HIV patients should be on separate wards, OR = 3.6, 95% CI (1.9–7.0). HCWs should be protected and encouraged to access HCT services in order to be effective role models in the prevention of HIV/AIDS.

## Introduction

Acquired immunodeficiency syndrome (AIDS) is one of the world’s most serious public health concerns, and poses an enormous challenge to most countries, especially developing countries such as Nigeria. Nigeria carries the second heaviest burden of HIV in Africa and has an expanding population of people living with HIV/AIDS (PLWHA) (Federal Republic of Nigeria, 2012). The first HIV Sentinel Survey in Nigeria in 1991 showed a prevalence of 1.8%. Subsequent sentinel surveys produced prevalence of 3.8% (1993), 4.5% (1996), 5.4% (1999), 5.8% (2001), 5.0% (2003), 4.4% (2005), 4.6% (2008) and 4.1% (2010) (Federal Republic of Nigeria, 2012). Health care workers (HCWs) are key players in the management of HIV infection because it is through the health infrastructure that individuals can learn of their HIV status, obtain prevention education, and seek treatment and care (Kiragu et al., 2007). The attitude of HCWs influences the willingness and ability of PLWHA to access care and influences the quality of care they receive (Kermode, Holmes, Langkham, Thomas, & Gifford, 2005). Discrimination against HIV-positive patients has profound impact on the care and support required for their optimal management particularly in resource-constrained settings (Sadoh, Fawole, Sadoh, Oladimeji, & Sotiloye, 2006). Stigmatization and discrimination impact negatively on interventions and act as barriers to adherence to antiretroviral therapy among PLWHA (Omosanya, Elegbede, Agboola, Isinkaye, & Omopariola, 2013). PLWHAs in Nigeria have been found to be subject to discrimination and stigmatization in the workplace, by family and communities (Reis et al., 2005) and may also face discrimination from HCWs (Famoroti, Fernandes, & Chima, 2013; Omosanya et al., 2013). Discriminatory or unethical behavior may hinder effective prevention and treatment by discouraging individuals from being tested or seeking information on how to protect themselves and others from HIV/AIDS.

This may affect government efforts at curtailing the spread of HIV/AIDS (Famoroti et al., 2013; Reis et al., 2005). Several studies on HCWs attitude to care of HIV patients have been reported from other countries. Anderson, Zheng, Wu, Li, and Liu (2003) reported that 24% of hospital-based health care professionals in China expressed reservations about caring for HIV infected patients. Hesketh, Duo, Li, and Tomkins (2005) reported that 30% of Chinese HCWs were not willing to treat people with HIV/AIDS and 81% would prefer not to. Similarly, about half of Iranian nursing staff were unwilling to care for patients with HIV/AIDS (Askarian, Hashem, Jaafari, & Assadian, 2006) and 84% of Jordanian nurses had negative attitudes towards them (Hassen & Wahsheh, 2011).

In Poland, 56% of doctors and 65% of nurses supported HIV testing of all inpatient admissions(Gañczak, 2007) and 86% of HCWs in India agreed with pre-operative HIV testing (Mahindra et al., 2007). Half of HCWs in a study carried out in South Africa reported they had observed or experienced patients being required to undergo HIV tests before surgery (Famoroti et al., 2013). A study done in Abeokuta, Nigeria reported that 13.9% of trained nurses were unwilling to take vital signs and carry out physical examination on HIV-positive patients (Sadoh et al., 2006). However, the attitude of other categories of HCWs in a tertiary hospital apart from doctors and nurses have not been explored. HCWs may become infected with HIV as a result of their personal sexual behavior. In addition to risks within the community within which they live, health service personnel face additional occupational risks from handling non-sterile injecting equipment or accidental exposure to blood or blood products. The frequency of exposure to blood and body fluids (BBFs) among HCWs varies according to occupation, procedures performed and use of preventive measures (Braczkowska et al., 2010; Hadadi, Afhami, Karbakhsh, & Esmailpour, 2008; Leszczyszyn-Pynka, Kłys-Rachwalska, Sacharczuk, & Boroń-Kaczmarska, 2004). Nurses were reported to have the most exposure in Poland (Braczkowska et al., 2010; Leszczyszyn-Pynka et al., 2004) and Taiwan (Hsieh, Chiu, Lee, & Huang, 2006).

In view of the risk of occupational exposure to HIV, it is important for HCWs to go for HIV counselling and testing (HCT). This is more so because they serve as source of information on HIV/AIDS and play a key role in mobilizing clients for HCT. HCT is a critical point to prevention, care, support and treatment for all people, and particularly for those already infected and affected. Tarwireyi and Majoko (2003) reported that 87.4% of HCWs in Zimbabwe had not gone for HCT and 77% did not want it done for reasons such as not being able to cope with the result, not having enough courage to go for the test and not wanting to do the test since HIV/AIDS had no cure. Similarly, 33% of HCWs in Zambia had ever been tested for HIV (Kiragu et al., 2007). In Ilorin, Nigeria 41.4% of HCWs had ever been screened for HIV infection and 65.2% of them were willing to know their status (Araoye, Salami, & Sekoni, 2004). In another study conducted among health workers in Ilorin, only 16.1% had ever been tested for HIV and psychological worry was the main reason for not going for HCT (Akande, 1999).

HCWs are expected to mobilize clients for HCT but if they are unwilling to take the test themselves they may be ineffective in this role. The University College Hospital (UCH) in Ibadan is the foremost tertiary health facility and a major referral center for other tertiary facilities in Nigeria. It has various cadres of HCWs and trainees and a WHO reference laboratory for HIV. The proportion of HCWs who have been tested for HIV is unknown. There is little data on UCH HCWs’ willingness to participate in HCT. Other studies addressing attitudes of HCWs to HIV-positive patients in Nigeria have focused on nurses. Little information is available about the attitude of doctors, laboratory scientists and ancillary staff such as porters and hospital maids.

The aim of this study was to determine occupational exposure and attitude to HIV-positive patients among HCWs at UCH, Ibadan. In addition, their level of uptake of HCT services and reasons for not having HCT done were also assessed.

## Methods

The study was carried out at University College Hospital (UCH), in Ibadan, Southwestern Nigeria. The hospital was established in 1957. It has 53 service and clinical departments and runs 75 consultative outpatient clinics per week in 45 specialty and sub-specialty disciplines. The hospital has 31 wards and a total bed capacity of 817. Inpatients admissions exceed 10,000 while outpatient clinic attendance approximate to over 17,000 a year. There is an antiretroviral clinic located within the hospital.

The study design was cross-sectional. The study population comprised all HCWs in the hospital. HCWs, as defined in this study, are clinical and other staff who have regular clinical contact with patients. These include doctors, dentists and nurses, paramedical professionals such as laboratory scientists, physiotherapists, radiographers and porters.

A total sampling of all health workers who gave informed consent were recruited for the study.

Semi-structured, self-administered questionnaires developed from the reviewed literature were used to collect information on socio-demographic characteristics, occupational characteristics, risk of exposure to HIV, attitude of HCWs to PLWHA, willingness to participate in HCT, factors affecting HCT uptake, and barriers to HCT uptake. The questionnaire was pretested among HCWs in Ladoke Akintola Teaching Hospital, another teaching hospital in Southwest Nigeria. Ethical approval to carry out the study was obtained from the Joint University of Ibadan and University College Hospital Institutional Review Committee and informed consent was obtained from respondents.

The heads of all clinical departments and unions of various categories of HCWs were informed about the study. In each department, the staff lists were obtained and used to distribute questionnaires to staff. Departmental secretaries distributed and collected the questionnaires from staff members in their respective departments.

All departments whose doctors are members of the College of Surgeons were listed as Surgical departments. Departments whose doctors are members of the College of Physicians were listed as Medical departments except laboratory departments which were categorized as Laboratory departments. Nursing staff were categorized as Nursing department. Ward maids, surgical attendants, dental hygienist, porters were categorized as other departments.

The data were analyzed with SPSS (Statistical Package for Social Sciences) version 15. Univariate analysis was done by generating frequencies of the variables and bivariate analysis was done using chi-square test. Level of significance was set at *p* < .05.

## Results

Table 1 shows socio-demographic characteristics of respondents. A total of 1500 questionnaires were administered and 977 completed questionnaires were returned giving a response rate of 65.1%. Mean age of respondents was 35.0 ± 8.4 years. Two-thirds were aged below 40 years. About two-thirds were females (69.7%) and ever married (65.7%). Almost all (96.9%) had tertiary education. Majority (75.1%) were doctors and nurses.

**Table 1.** Socio-demographic characteristics of respondents.

Socio-demographic characteristics	*n* (%) *N* = 977
Age group (years)	
<40	596 (61.0)
≥40	214 (21.9)
Missing	167 (17.1)
Mean Age (±SD) years	35.0 (±8.4)
Sex	
Male	296 (30.3)
Female	681(69.7)
Marital status	
Never married	335(34.3)
Ever married/cohabiting	642 (65.7)
Level of education	
None	4 (0.4)
Primary	9 (0.9)
Secondary	18 (1.8)
Tertiary	943(96.9)
Religion	
Christianity	872(89.3)
Islam	97 (9.9)
Others	8 (0.8)
Occupation	
Nurses	405 (41.5)
Doctors	328 (33.6)
Lab scientists	78 (8.0)
Others^a^	126 (12.9)
Missing	40 (4.0)

^a^Ward maids, surgical attendants, dental hygienist, and porters.

Table 2 shows the responses on perception of exposure to HIV at work and experience of workplace accidents with exposure to BBFs. Majority of respondents reported that their jobs involved exposure to BBFs (92.3%), use of needles (88.1%) and direct care of patients (87.2%). Staff from the nursing department reported the highest exposure to BBFs (98.9%) and use of needles (97.8%) compared to other departments (*p* < .05). Respondents in the nursing department (96.7%) reported highest involvement in the direct care of patients (*p* < .05). Two-thirds of respondents (67.7%) felt they were adequately protected against HIV infection in the workplace. Respondents in the medical (56.9%) and surgical (56.9%) departments reported the lowest level of protection in the work place (*p* < .05). Forty percent of respondents reported that their risk of contracting HIV at work was high. A higher proportion of respondents from surgical departments (49.7%) reported a high risk of contracting HIV at work, followed by those in the laboratory departments (41.3%) (*p* < .05).

**Table 2.** Risk of exposure and workplace accidents of HCWs by departments.

Risk of exposure	Medical departments *n* (%)	Surgical departments *n* (%)	Laboratory departments *n* (%)	Nursing departments *n* (%)	Other departments^a^*n* (%)	Total *n* (%)	*p* Value
Risk of exposure of HCWs							
Job involves exposure to BBFs	170 (93.4)	185 (98.4)	94 (87.9)	365 (98.9)	60 (59.4)	874 (92.3)	.000
Job involves use of needles	167 (92.3)	184 (97.4)	98 (90.7)	359 (97.8)	26 (25.5)	834 (88.1)	.000
Job involves direct care of patient	153 (84.5)	175 (93.6)	71 (67.6)	356 (96.7)	65 (65.7)	820 (87.2)	.000
Feel adequately protected in the work place	103 (56.9)	107 (56.9)	72 (70.6)	276 (75.6)	76 (75.2)	634 (67.7)	.000
High risk of contracting HIV at work	73 (40.1)	96 (49.7)	45 (41.3)	149 (40.9)	17 (16.7)	380 (40.0)	.000
Workplace accidents in the last one year with potential risk of infection							
Accidental exposure in the last one year	100 (55.9)	115 (60.2)	36 (33.0)	154 (43.0)	35 (35.4)	440 (47.0)	.000
Splash of BBFs on intact skin	81 (77.1)	88 (77.2)	26 (40.6)	125 (64.4)	11 (22.0)	331 (62.8)	.000
Needle stick injury	61 (57.5)	64 (54.7)	17 (25.8)	69 (37.1)	11 (20.8)	220 (42.0)	.000
Cuts from broken bottles	19 (20.7)	30 (29.7)	5 (8.5)	53 (31.0)	7 (14.3)	114 (24.2)	.002
Splash of BBFs on mucosal lining	19 (20.2)	44 (40.7)	6 (10.3)	30 (18.1)	5 (10.2)	104 (21.9)	.000
Splash of BBFs on open wound	11 (12.1)	22 (21.4)	2 (3.4)	33 (19.5)	9 (18.8)	77 (16.4)	.000
Cuts from scalpel	6 (7.3)	18 (19.8)	3 (5.4)	27 (16.7)	3 (6.4)	57 (13.0)	.013

^a^Other departments include ward maids, surgical attendants, dental hygienist and porters.

About half (47.0%) of respondents had accidental exposure to BBFs in the year preceding the study. Types of exposure most commonly reported were splash of BBFs on intact skin (62.8%) and needlestick injuries (42.0%). Setting of intravenous lines (19.5%), surgical operations (15.9%) and recapping of needles (15.0%) were the most commonly reported activities being carried out during exposure to BBFs. Accidental exposure in the last one year was mostly reported by those in the surgical departments (60.2%). Cuts from broken bottles were mostly reported by staff from the nursing department (31.3%) (*p* < .05).

Table 3 shows the attitude of HCWs to HIV-positive patients; attitude about administrative issues with regard to HIV and attitude to HCT. Majority (99.4%) of respondents believed that the quality of life of patients with HIV/AIDS can be improved with counselling. Very few (7.9%) will however refuse to treat a patient with HIV/AIDS to protect themselves and their families. Half of the staff from the laboratory departments believed that people with HIV/AIDS should be on a separate ward in a hospital or clinic. Majority of respondents believed that all obstetric (91.2%) and surgical (89.2%) patients should be routinely tested for HIV on admission to hospital. A higher proportion of nurses than other staff believed that all patients should be routinely tested for HIV on admission to hospital (*p* < .05). Very few respondents (26.6%) reported that charts/beds of patients with HIV/AIDS should be marked so clinic/hospital workers can be aware and 23.7% felt that health professionals with HIV/AIDS should not work in any area of health care that requires patient contact.

**Table 3.** Attitude of HCWs to HIV-positive patients, HIV/AIDS and routine HIV testing.

Attitude of HCWs	Medical depts *n* (%)	Surgical depts *n* (%)	Laboratory depts *n* (%)	Nursing depts *n* (%)	Other depts *n* (%)	Total *n* (%)	*p* Value
Attitude of HCWs to HIV-positive patients							
The quality of life of patients with HIV/AIDS can be improved with counselling	184 (100)	190 (99.0)	111 (100)	303 (99.2)	100 (99.0)	948 (99.4)	.42
People with HIV/AIDS should be on a separate ward in a hospital or clinic.	48 (26.4)	48 (25.1)	54 (50.0)	86 (23.4)	46 (46.0)	282 (29.7)	.000
Can refuse to treat a patient with HIV/AIDS to protect self and family	22 (12.0)	19 (9.9)	4 (3.7)	21 (5.8)	9 (8.9)	75 (7.9)	.000
Attitude of HCWs to HIV /AIDS							
All obstetric patients should be routinely tested for HIV on admission to hospital	161 (88.0)	176 (92.1)	94 (87.9)	346 (95.6)	85 (83.3)	862 (91.2)	.002
Staff and health care professionals should be told when a patient has HIV/AIDS so they can protect themselves	159 (18.7)	173 (20.3)	92 (10.8)	336 (39.4)	92 (10.8)	852 (89.8)	.218
All surgical patients should be routinely tested for HIV on admission to hospital	157 (85.8)	164 (86.8)	96 (87.3)	337 (93.1)	90 (88.2)	844 (89.2)	.158
All patients should be routinely tested for HIV on admission to hospital	106 (58.2)	104 (54.5)	70 (64.2)	291 (81.1)	69 (68.3)	640 (67.9)	.000
There are instances where it is appropriate to test for a patient for HIV/AIDS without the patient’s knowledge or permission	111 (61.7)	112 (58.3)	56 (50.0)	205 (56.8)	56 (55.4)	540 (57.1)	.016
The charts/beds of patients with HIV/AIDS should be marked so clinic/hospital workers can know	36 (19.7)	68 (35.4)	29 (26.9)	81 (22.3)	38 (37.6)	252 (26.6)	.000
A health professional with HIV/AIDS should not be working in any area of health care that requires patient contact	33 (18.1)	47 (24.9)	26 (23.9)	90 (25.3)	2/;;6 (25.5)	222 (23.7)	.001
Attitude to routine HCT of HCWs							
All prospective health care workers should submit to mandatory HCT	92 (50.5)	108 (56.8)	70 (63.6)	236 (65.4)	69 (67.6)	575 (60.8)	.006
HIV screening should not be performed on health workers	19 (10.5)	39 (21.0)	18 (18.8)	48 (14.2)	19 (20.7)	143 (16.0)	.012
HCT should be part of pre-employment medical examination for health workers	104 (58.1)	109 (58.6)	64 (67.4)	230 (67.6)	57 (60.6)	564 (63.1)	.073
All dentists and surgeons should be tested for HIV	120 (67.4)	116 (62.0)	72 (73.5)	232 (68.6)	63 (65.6)	603 (67.2)	.611
Regular HCT should be performed on health workers at least once a year	110 (61.8)	106 (57.6)	68 (72.3)	237 (70.7)	65 (67.7)	586 (66.1)	.094

About two-thirds of respondents (60.8%) believe that all prospective HCWs should submit to mandatory HCT, 63.1% felt that HCT should be part of pre-employment medical examination for health workers and 66.1% felt that regular HCT should be performed on health workers at least once a year.

Table 4 shows HCWs’ practice with regard to HIV-positive patients and participation in HCT. Very few respondents (3.8%) reported they had refused to care for a patient with HIV/AIDS, verbally mistreated a patient with HIV/AIDS (4.1%), and gave confidential information to a non-family member (10.6%) and family member (12.0%).

**Table 4.** HCWs’ behavior towards HIV-positive patient and participation in HCT.

HCWs’ practice	Medical depts. *n* (%)	Surgical depts *n* (%)	Laboratory depts *n* (%)	Nursing depts *n* (%)	Other depts *n* (%)	Total *n* (%)	*p* Value
Refused a patient with HIV/AIDS admission	7 (3.9)	6 (3.1)	4 (3.7)	5 (1.4)	1 (1.1)	23 (2.5)	.000
Observed others refuse a patient with HIV/AIDS admission	39 (21.5)	32 (16.7)	13 (12.0)	33 (9.4)	10 (10.6)	127 (13.7)	.000
Refused to care for a patient with HIV/AIDS	7 (3.9)	11 (5.7)	4 (3.7)	10 (2.8)	3 (3.2)	35 (3.8)	.000
Observed others refuse to care for a patient with HIV/AIDS	64 (35.4)	67 (35.3)	20 (18.3)	62 (17.7)	15 (16.1)	228 (24.7)	.000
Verbally mistreated a patient with HIV/AIDS	6 (3.3)	8 (4.2)	4 (3.7)	16 (4.5)	4 (4.3)	38 (4.1)	.000
Observed others verbally mistreat a patient with HIV/AIDS	55 (30.4)	49 (25.7)	18 (16.5)	70 (20.2)	14 (15.4)	206 (22.4)	.000
Gave confidential information to a family member	22 (12.4)	26 (13.8)	15 (14.4)	37 (10.7)	9 (10.0)	109 (12.0)	.000
Observed others give confidential information to a family member	51 (28.5)	54 (28.3)	26 (24.8)	60 (17.2)	14 (15.4)	205 (22.4)	.000
Gave confidential information to a non-family member	19 (10.5)	29 (15.2)	8 (7.6)	30 (8.6)	11 (12.1)	97 (10.6)	.000
Observed others give confidential information to a non-family member	32 (17.7)	41 (21.4)	12 (11.7)	42 (12.0)	19 (20.7)	146 (15.9)	.000
HCWs’ participation in HCT							
Ever gone for HCT	120 (66.3)	91 (48.7)	41 (41.0)	185 (54.7)	40 (42.1)	477 (52.9)	.000
Ever provided HCT information	155 (86.6)	125 (67.9)	58 (58.6)	241 (72.2)	49 (51.6)	628 (70.5)	.000
Referred any for HCT	141 (78.3)	99 (53.5)	57 (57.0)	186 (56.0)	33 (34.7)	516 (57.8)	.000

Eighty percent of respondents had ever heard of HCT and 65% were aware of an HCT center in UCH. About half of respondents (52.9%) had gone for HCT themselves and two-thirds (66.0%) of these because they wanted to know their status. Among those who had not gone for HCT, 68.6% of them were willing to go. Lack of confidentiality (8.5%), not perceived as being at risk (7.5%), not interested in knowing (6.8%) and lack of social security for HIV-positive workers (5.4%) were some of the reasons for unwillingness to go for HCT. Majority of respondents (70.5%) had ever provided HCT information to patients and 57.8% had ever referred anyone for HCT. A higher proportion of respondents in Medical departments than other departments had ever gone for HCT (66.3%), provided HCT information (86.6%) and referred any for HCT (78.3%) (*p* = .000).

### Factors associated with accidental exposure to BBFs, attitude to HIV patients and HCT.

Table 5 shows bivariate analysis of socio-demographic characteristics of HCWs and accidental exposure to BBFs in the last one year, attitude to HIV patients and HCT uptake. Factors associated with accidental exposure in last one year were age <40 years (*p* = .004), being in medical, surgical or nursing departments (*p* = .000) and having spent less than five years at work (*p* = .02). Working in laboratory departments (*p* = .000) and having spent less than five years at work (*p* = .02) were associated with poor attitude of HCWs to HIV patients in terms of segregation on separate ward. The only factor associated with poor attitude of HCWs to HIV patients in terms of mandatory HIV test for all patients was being in the nursing department (*p* = .000).

**Table 5.** Bivariate analysis of socio-demographic characteristics and accidental exposure to BBFs, attitude to HIV patients and HCT Uptake.

	Outcome variables			
Socio-demographic characteristics	Accidental exposure to BBFs	HIV patients should be on separate wards	Routine HIV test for all patients	HCT uptake
Age group(years)				
<40	290 (50.7)*	177 (30.3)	383 (65.9)	314 (56.0)*
≥40	77 (37.6)	52 (25.0)	147 (70.7)	90 (47.2)
Department				
Medical	100 (55.9)**	48 (26.4)**	106 (58.2)**	120 (66.3)**
Surgical	115 (60.2)	48 (25.1)	104 (54.5)	91 (48.7)
Nursing	154 (43.0)	86 (23.4)	291 (81.1)	185 (54.7)
Others	35 (35.4)	46 (46.0)	69 (68.3)	40 (42.1)
Laboratory	36 (33.0)	54 (50.0)	70 (64.2)	41 (41.0)
Length of years at work				
≤5	168 (52.0)*	107 (32.7)*	214 (66.0)	162 (51.3)
>5	193 (44.1)	112 (24.9)	296 (66.7)	242 (57.3)

*p < .05.

**p < .001.

### Predictors of accidental exposure to BBFs in the last one year, attitude to HIV patients and HCT

Logistic regression analyses of factors significantly associated with accidental exposure to BBFs in the last one year, attitude to HIV patients and HCT were done. Table 6 shows that the predictors of accidental exposure to BBFs in the last year. The main predictor of accidental exposure in the last year was working in surgical department, OR = 1.7, 95% CI (1.1–2.6). HCWs aged <40 years, OR = 5.5, 95% CI (1.9–15.9), who had worked for greater than five years, OR = 3.6, 95% CI (1.4–9.3) and working in nursing department, OR = 6.8, 95% CI (1.7–27.1) were more likely to report exposure to BBFs. Respondents in the nursing departments were more likely to require routine HIV test for all patients, OR = 3.9, 95% CI (2.4–6.2). HCWs in the laboratory departments were more likely to believe that HIV patients should be on separate wards compared to those in the medical departments OR = 3.6, 95% CI (1.9–7.0). Predictors for HCT uptake were age <40 years, OR = 1.6, 95% CI (1.1–2.4), having worked for greater than five years, OR = 1.5, 95% CI (1.03–2.2) and working in medical department, OR = 1.7, 95% CI (1.1–2.8).

**Table 6.** Logistic regression analysis of socio-demographic and accidental exposure to BBFs, attitude to HIV patients and HCT uptake.

	Outcome variables			
Socio-demographic characteristics	Accidental exposure to BBFs in last one year	HIV patients should be on separate wards	Routine HIV test for all patients	HCT uptake
Age group (years)				
<40	1.2 (0.8–1.8)	1.2 (0.7–1.8)	–	1.6 (1.1–2.4)*
≥40	1	1		1
Department				
Medical	2.1 (1.1–3.9)*	0.3 (0.1–0.5)**	0.8 (0.4–1.5)	2.0 (1.1–4.0)*
Surgical	2.7 (1.4–5.2)*	0.3 (0.2–0.6)**	0.7 (0.4–1.3)	1.0 (0.5–1.9)
Nursing	1.6 (0.9–2.9)	0.3 (0.2–0.6)**	3.0 (1.6–5.6)*	1.2 (0.6–2.1)
Others	0.7 (0.3–1.6)	0.6 (0.3–1.2)	1.2 (0.6–2.5)	0.9 (0.4–1.9)
Laboratory	1	1	1	1
Length of years at work				
≤5	1.3 (0.9–1.9)	1.3 (0.9–2.0)	–	–
>5	1	1		

*p < .05.

**p < .001.

## Discussion

Majority of HCWs reported that their jobs involved exposure to BBFs. Setting of intravenous lines, surgical operations and recapping of needles were the most commonly reported activities being carried out during exposure to BBFs. This is similar to the findings of other studies which reported that recapping of needles (Leszczyszyn-Pynka et al., 2004), performing surgical procedures (Lal, Meghachandra, Singh, Malhotra, & Ingle, 2006), handling surgical equipment (Gańczak, Szych, & Karakiewicz, 2012), handling medical wastes (Rapparini et al., 2007), disposal into sharp containers (Do et al., 2003) and blood withdrawal (Muralidhar, Singh, Jain, Malhotra, & Bala, 2015) were the common procedures leading to exposure to BBFs. Nurses reported the highest use of needles and exposure to BBFs. This is similar to findings by Hsieh et al. (2006) and Hadadi et al. (2008) and is probably because needles are used mostly by nurses (WHO, 2014a). About half of HCWs reported accidental exposure to BBFs in the last year with staff in the surgical departments reporting the highest accidental exposures, the highest number of splash of BBFs on intact skin and open wounds, needle stick injuries and cuts from scalpel. Surgical staff were three times more likely to experience accidental exposure to BBFs than those in the Laboratory departments. Surgeons have contact with BBFs of patients during surgical operations and non-availability of relevant devices has been reported as the most important factor militating against the use of universal precautions by surgeons in developing countries (Olapade-Olaopa, Salami, & Afolabi, 2006). Splash of BBFs on intact skin was the most commonly reported exposure. Other reports by WHO (2014a), Braczkowska et al. (2010), Davanzo, Frasson, Morandin, and Trevisan (2008) and Lal et al. (2006) indicated that needle stick injuries were the commonest type of exposure to BBFs. This is probably because these studies were conducted among doctors and nurses alone while our study included other groups of health workers such as laboratory scientists, laboratory attendants and ward maids who do not frequently use needles. The high frequency of exposure by splash of BBFs on intact skin in this population constitutes a significant risk as it can be a source of infection for Ebola virus disease among health care workers (WHO, 2014b).

One-third of respondents reported they were not adequately protected from exposure to BBFs in the workplace. This is of great concern especially in the surgical departments where the highest risk of contracting HIV at work and the lowest level of protection were reported. In order to mitigate this risk, known strategies to protect workers and prevent exposure such as standard precautions, immunization against hepatitis B, provision of personal protective equipment and management of exposures should be adhered to.

HCWs in this study had positive attitudes towards PLWHA. Only few respondents will mark charts/beds of patients with HIV/AIDS and 24% believed health professionals with HIV/AIDS should not work in any area of health care that requires patient contact. This proportion is lower than 40% of health care professionals in health care facilities in four Nigerian states who believed that health professional with HIV/AIDS should not work in any area of health care that requires patient contact (Reis et al., 2005). Very few, 7.9%, would refuse to treat patients with HIV/AIDS. This is similar to 9% of health care professionals in health care facilities in four Nigerian states who would refuse to care for PLWHA (Reis et al., 2005). However, a higher proportion (15%) of HCWs in Abeokuta were unwilling to take vital signs and carry out physical examination on PLWHA (Sadoh et al., 2006).

HCWs in laboratory departments were more likely than those in the medical department to want HIV patients to be admitted on separate wards. HCWs in the laboratory departments are frequently involved in handling of specimens of BBFs and have a high level of perceived risk of contracting HIV. This may be the reason why they have this unfavorable attitude. This is a discriminatory attitude that could affect patient’s attendance at health centers for obtaining ARV and regular medical check-ups or adherence of patients to treatment plans (Famoroti et al., 2013; Omosanya et al., 2013). Though majority of HCWs will not refuse to treat PLWHA, 90% of them would want patients to be screened prior to surgical and obstetric procedures. This is similar to findings among HCWs in India where 90% of HCWs endorsed the practice of conducting mandatory HIV testing prior to surgery (Mahindra et al., 2007).

A higher proportion of nurses believed all patients should be routinely screened for HIV. Nurses have been reported to have a less favorable attitude to PLWHA. Similarly, in India, doctors were reported to be less prejudiced when compared to nurses in their attitude towards PWLHA (Mahindra et al., 2007).

Trained nurses and auxiliary nurses compared with physicians in Nigeria were reported to be more likely to deny patient services based on HIV sero-status (Sadoh et al., 2006). Similarly, a higher proportion of nurses than other HCWs in China tended to avoid contact with PLWHA and more than two-thirds of Jordanian nurses (84%) refused to provide care to patients who tested positive for HIV/AIDS (Hassen & Wahsheh, 2011; Lin, Li, Wan, Wu, & Yan, 2012). This unfavorable attitude to PLWHA could be because nurses are more involved in the direct care of and contact with patients and their perceived risk of contracting HIV at work is high (Famoroti et al., 2013). Health care providers have been reported to perceive HIV testing of patients as a self-protection strategy out of fear of infection risk at work. Furthermore, fear of contracting HIV and death from HIV infection has been reported as factors related to HIV stigma in health care (Li et al., 2007). Mandatory HIV testing of patients is controversial. Proponents of mandatory HIV testing believe it ensures early detection and treatment of infected individuals which is beneficial while some believe it is ethically wrong as it infringes on patients’ confidentiality if they are not informed that results will be shared (Li et al., 2007). In addition, patients at risk of HIV may not seek medical care in the hospital if they know they would be routinely screened which may lead to other public health problems. Presently in UCH, routine HIV testing is only done for patients going for surgical or gynecological operations.

Two-thirds of respondents believe HCT should be part of pre-employment medical examination for all prospective health care workers and that they should submit to mandatory HCT, at least once a year. Mandatory HIV testing has been introduced for a large number of HCWs in the developed world (Salkeld & McGeehan, 2010). Salkeld, McGeehan, Chaudhuri, and Kerslake (2009) reported that junior doctors who were offered an HIV test as part of their pre-employment occupational health checks by National Health Service Trusts in the United Kingdom were highly critical of the manner the HIV test was offered. In UCH, HCT is prohibited for recruitment and employment purposes (University College Hospital/College of Medicine, 2005)

In this study, majority of respondents had heard of HCT and were aware of an HCT center in UCH but only half had gone for HCT at the time of this study. HCWs in the medical departments were twice more likely to have gone for HCT than those in the laboratory departments. Though HCWs in the laboratory departments reported lowest direct contact with patients they are at risk of exposure to BBFs and should therefore be encouraged to go for HCT. Majority of respondents had also provided HCT information to patients and some had referred patients for HCT. It is imperative for HCWs to be role models. They should not only provide information on HCT to patients but also undergo the test themselves.

## Limitations of study

A selection bias could have occurred in the participation of the HCWs entering the study with more educated respondents being more willing to participate. However, efforts were made to follow up and retrieve questionnaires from all cadres of HCWs. It is possible that respondents could have copied other HCWs’ questionnaires but this would have happened mainly within departments and would not affect the results significantly. This study did not assess knowledge and training of HCWs on HIV/AIDS which could have influenced their attitude to PLWHA. Studies have shown that HCWs with knowledge and training of HCWs on HIV/AIDS were less likely to stigmatize and discriminate PLWHA (Adetoyeje, Bashir, & Ibrahim, 2007; Famoroti et al., 2013).

## Conclusion

The study concludes that splash of BBFs on intact skin was the most commonly reported exposure among HCWs in this tertiary hospital. HCWs in surgical departments reported they were least protected. Majority of HCWs had positive attitude towards PLWHA. However, a large proportion of nurses believed that all patients should be routinely screened for HIV. Half of HCWs had accessed HCT service. There is need to ensure that workers are well protected in the workplace. All aspects of Standard Precautions should be employed in all patient care whether the patient is HIV positive or not and protective equipment required to practice Standard Precaution should always be available to reduce the risk of exposure to BBFs. The University College Hospital workplace policy on HIV/AIDS should be made available to HCWs so they can be aware of what is expected of them in their care of PLWHA. HCWs should be encouraged to go for HCT in order to be effective role models in the prevention of HIV/AIDS.

## References

[CIT0001] Adetoyeje Y. O., Bashir O. O., & Ibrahim S. B. (2007). Physicians and HIV and does knowledge influence their attitude and comfort in rendering care. African Journal of Health Sciences, , 37–43.

[CIT0002] Akande T. (1999). Willingness of health workers to undergo HIV screening. Nigerian Quarterly Journal of Hospital Medicine, (4), 303–306.

[CIT0003] Anderson A. F., Zheng Q., Wu G., Li Z., & Liu W. (2003). Human immunodeficiency virus knowledge and attitudes among hospital-based healthcare professionals in Guangxi Zhuang autonomous region, People’s Republic of China. Infection Control & Hospital Epidemiology, (2), 128–31. doi: 10.1086/502171 12602695

[CIT0004] Araoye M., Salami A., & Sekoni O. (2004). Occupational risk of exposure to HIV/AIDS and Willingness to know status among health workers Nigerian. Journal of Genito-Urinary Medicine, (1&2), 26–30.

[CIT0005] Askarian M., Hashem I. Z., Jaafari P., & Assadian O. (2006). Knowledge about HIV infection and attitude of nursing staff toward patients with AIDS in Iran. Infection Control & Hospital Epidemiology, (1), 48–53. doi: 10.1086/500002 16418987

[CIT0006] Braczkowska B., Kowalska M., Beniowski M., Zejda J., Mazur W., & Witor A. (2010). Occupational exposure to HIV in health care workers. Silesia Voivodeship Medycyna Pracy, (3), 315–322. 20677431

[CIT0007] Davanzo E., Frasson C., Morandin M., & Trevisan A. (2008). Occupational blood and body fluid exposure of university health care workers. American Journal of Infection Control, (10), 753–756. doi: 10.1016/j.ajic.2008.04.254 18945522

[CIT0008] Do A. N., Ciesielskim C. A., Metler R. P., Hammett T. A., Li J., & Fleming P. L. (2003). Occupationally acquired human immunodeficiency virus (HIV) infection: National case surveillance data during 20 years of the HIV epidemic in the United States. Infection Control & Hospital Epidemiology, (2), 86–96. doi: 10.1086/502178 12602690

[CIT0009] Famoroti T. O., Fernandes L., & Chima S. C. (2013). Stigmatization of people living with HIV/AIDS by healthcare workers at a tertiary hospital in KwaZulu-Natal, South Africa: A cross-sectional descriptive study. BMC Medical Ethics, (Suppl 1), S6. doi: 10.1186/1472-6939-14-S1-S6 24564982 PMC3878339

[CIT0010] Federal Republic of Nigeria. Global AIDS response. Country Progress Report. NACA (2012).

[CIT0011] Gañczak M. (2007). Stigma and discrimination for HIV/AIDS in the health sector: A Polish perspective. Revista interamericana De Psicologia = Interamerican Journal of Psychology, , 57–66.

[CIT0012] Gańczak M., Szych Z., & Karakiewicz B. (2012). Assessment of occupational exposure to HBV, HCV and HIV in gynecologic and obstetric staff. Medycyna Pracy, (1), 11–17. 22774460

[CIT0013] Hadadi A., Afhami S., Karbakhsh M., & Esmailpour N. (2008). Occupational exposure to body fluids among healthcare workers: A report from Iran. Singapore Medical Journal, (6), 492–496. 18581025

[CIT0014] Hassen Z. M., & Wahsheh M. A. (2011). Knowledge and attitudes of Jordanian nurses towards patients with HIV/AIDS: Findings from a nationwide survey. Issues in Mental Health Nursing, (12), 774–784. doi: 10.3109/01612840.2011.610562 22077750

[CIT0015] Hesketh T., Duo L., Li H., & Tomkins A. (2005). Attitudes to HIV and HIV testing in high prevalence areas of China: Informing the introduction of voluntary counseling and testing programme. Sexually Transmitted Infections, , 108–112. doi: 10.1136/sti.2004.009704 15800085 PMC1764667

[CIT0016] Hsieh W., Chiu N., Lee C., & Huang F. (2006). Occupational blood and infectious body fluid exposures in a teaching hospital-a three-year review. Journal of Microbiology Immunology and Infection, , 321–327. 16926979

[CIT0017] IBM: Chicago: SPSS Inc (2006). SPSS Statistics for Windows, Version 15.0. Released.

[CIT0018] Kermode M., Holmes W., Langkham B., Thomas M., & Gifford S. (2005). HIV related knowledge, attitudes & risk perception amongst nurses, doctors & other healthcare workers in rural India. The Indian Journal of Medical Research, (3), 258–264. 16251785

[CIT0019] Kiragu K., Ngulube T., Nyumbu M., Njobvu P., Eerens P., & Mwaba C. (2007). Sexual risk-taking and HIV testing among health workers in Zambia. AIDS and Behavior, (1), 131–136. doi: 10.1007/s10461-006-9091-9 16779658

[CIT0020] Lal P., Meghachandra, Singh M., Malhotra R., & Ingle G. (2006). Perception of risk and potential occupational exposure to HIV/AIDS among medical interns in Delhi. Journal of Communicable Diseases, 38(4), 345–349. 17913211

[CIT0021] Leszczyszyn-Pynka M., Kłys-Rachwalska M., Sacharczuk B., & Boroń-Kaczmarska A. (2004). Occupational exposure to human immunodeficiency virus (HIV) – how can we reduce the risk? International Journal of Occupational Safety and Ergonomics, (4), 425–429. doi: 10.1080/10803548.2004.11076626 15598365

[CIT0022] Li L., Wu Z., Wu S., Lee S., Rotheram-Borus M., Detels R., … Sun S. (2007). Mandatory HIV testing in China: The perception of health-care providers. International Journal of STD & AIDS, (7), 476–481. doi: 10.1258/095646207781147355 17623506 PMC2800998

[CIT0023] Lin C., Li L., Wan D., Wu Z., & Yan Z. (2012). Empathy and avoidance in treating patients living with HIV/AIDS (PLWHA) among service providers in China. AIDS Care, (11), 1341–1348. doi: 10.1080/09540121.2011.648602 22292939 PMC3419277

[CIT0024] Mahindra V. S., Gilborn L., Bharat S., Modi R., Gupta I., George B., … Pulerwitz J. (2007). Understanding and measuring aids related stigma in health care settings: A developing country perspective. SAHARA-J: Journal of Social Aspects of HIV/AIDS, , 616–625. doi: 10.1080/17290376.2007.9724883 18071613

[CIT0025] Muralidhar S., Singh P., Jain R., Malhotra M., & Bala M. (2010). Needle stick injuries among health care workers in a tertiary care hospital of India. The Indian Journal of Medical Research, , 405–410. 20418554

[CIT0026] Olapade-Olaopa E. O., Salami M. A., & Afolabi A. O. (2006). HIV/AIDS and the surgeon. African Journal of Medicine and Medical Sciences, (Suppl), 77–83. 18050778

[CIT0027] Omosanya O. E., Elegbede O. T., Agboola S. T., Isinkaye A. O., & Omopariola O. A. (2013). Effects of stigmatization/discrimination on antiretroviral therapy adherence among HIV-infected patients in a rural tertiary medical center in Nigeria. Journal of The International Association of Providers of Aids Care, 1–4, doi: 10.1177/2325957413475482 23518308

[CIT0028] Rapparini C., Saraceni V., Lauria L., Barroso P., Vellozo V., Cruz M., … Durovni B. (2007). Occupational exposures to bloodborne pathogens among healthcare workers in Rio de Janeiro, Brazil. Journal of Hospital Infection, (2), 131–173. doi: 10.1016/j.jhin.2006.09.027 17178428

[CIT0029] Reis C., Heisler M., Amowitz L., Moreland R. S., Mafeni J., Anyamele C., … Benatar S. (2005). Discriminatory attitudes and practices by health workers toward patients with HIV/AIDS in Nigeria. PLoS Medicine, (8), e246. doi: 10.1371/journal.pmed.0020246 16022564 PMC1176239

[CIT0030] Sadoh A., Fawole A., Sadoh W., Oladimeji A., & Sotiloye O. (2006). Attitude of health care workers to HIV/AIDS. African Journal of Reproductive Health, (1), 39–46. doi: 10.2307/30032442 16999193

[CIT0031] Salkeld L., & McGeehan S. (2010). HIV testing of health care workers in England – a flawed policy. Journal of Health Services Research & Policy, , 62–67. doi: 10.1258/jhsrp.2009.009095 20147426

[CIT0032] Salkeld L., McGeehan S., Chaudhuri E., & Kerslake I. (2009). HIV testing of junior doctors: Exploring their experiences, perspectives and accounts. Journal of Medical Ethics, (7), 402–406. doi: 10.1136/jme.2008.027052 19567686

[CIT0033] Tarwireyi F., & Majoko F. (2003). Health workers’ participation in voluntary counseling and testing in three districts of Masholand, East Province, Zimbabwe. Central African Journal of Medicine, (5-6), 58–62. 15214284

[CIT0034] University College Hospital/College of Medicine (2005). *Workplace policy on HIV/AIDS* (*1st ed*). Ibadan: APIN-PMTCT, UCH-COM.

[CIT0035] WHO (2014a, November 12). Occupational health. Occupational health topics. Needlestick injuries. Protecting health-care workers. Preventing needle stick injuries. Retrieved from: http://www.who.int/occupational_health/topics/needinjuries/en/

[CIT0036] WHO (2014b, November 12). Occupational health. Occupational health topics. Health workers. Health worker occupational health. Retrieved from: http://www.who.int/occupational_health/topics/hcworkers/en/

